# Apoptosis Induction Pathway in Human Colorectal Cancer Cell Line SW480 Exposed to Cereal Phenolic Extracts

**DOI:** 10.3390/molecules24132465

**Published:** 2019-07-04

**Authors:** Shiwangni Rao, Kenneth Chinkwo, Abishek Santhakumar, Stuart Johnson, Christopher Blanchard

**Affiliations:** 1 School of Biomedical Sciences, Australian Research Council (ARC) Industrial Transformation Training Centre (ITTC) for Functional Grains, Graham Centre for Agricultural Innovation, Charles Sturt University, Wagga Wagga, NSW 2650, Australia; 2 Agriculture and Food Discipline, School of Molecular and Life Sciences, Curtin Health Innovation Research Institute, Curtin University, Perth, WA 6845, Australia

**Keywords:** apoptosis, cytotoxicity, colorectal cancer, polyphenols, antioxidant activity, chemopreventive

## Abstract

Cereal phenolic extracts have previously been investigated for their potential anticancer properties; however, the exact mechanisms involved in the inhibition of tumour growth are unclear. One possible mechanism is the induction of apoptosis which is characterised by cell shrinkage, protein fragmentation, and DNA degradation followed by rapid engulfment of cell debris by macrophages. This study examines the ability of phenolic extracts from four cereals: rice, barley, oats and sorghum to induce apoptosis on colorectal cancer cells SW480. Wholegrain extracts from pigmented varieties of red rice, purple rice, black sorghum, and brown sorghum showed a significant reduction in cancer cell proliferation. Morphological observation using APOPercentage™ dye indicated positive for apoptosis. Further analyses of Yunlu29 (rice), Shawaya Short Black 1 and IS1136 (sorghum) showed expression of p53 and confirmed activation of multiple caspases, specifically for caspase 3 and 7. Purple rice, on the other hand, did not upregulate caspase 3 and 7, hence, suggestive of cell cycle arrest. Therefore, phenolic compounds present in cereals such as pigmented rice and sorghum may suppress cancer cell proliferation through the activation of the apoptosis.

## 1. Introduction

Cancer is a multifaceted disease and, of its many forms lung, breast and bowel cancer are the most common globally [1]. According to the World Health Organization, a third of cancer-related deaths arise as a result of poor dietary patterns [2]. Consumption of foods with added functional benefits such as antioxidant activity has been associated with a reduction in the risk of cancer progression [3,4]

Research has shown that some whole grain varieties of cereals such as rice (*Oryza sativa* L.), barley (*Hordeum vulgare* L.), oats (*Avena sativa* L.) and sorghum (*Sorghum bicolor* L.) are good sources of phenolic compounds. These phenolic compounds are commonly found in the lipid rich layers of the bran and have the ability to readily scavenge free radicals [5,6]. Anthocyanins and proanthocyanidins are two major classes of bioactive phenolic compounds that have been identified in cereal grains, which are predominantly present in pigmented varieties. Derivatives of anthocyanin present in sorghum, 3-deoxyanthocyanidin have been demonstrated to have anti-proliferative potential [7,8,9]. In addition, avenanthramide, a unique phenolic alkaloid that is only found in oats, has also been identified as an active scavenger of free radicals in chemical assays and in vitro, with potential anti-cancer properties [10,11,12].

Apoptosis is a form of programmed cell death, where the externalization of phosphatidylserine (PS) alters cell membrane configuration and permeability. In addition, cells also undergo other morphological changes including cell shrinkage and DNA fragmentation. Apoptosis can be induced in compromised cells through the extrinsic (via the death receptor) or intrinsic (via the mitochondria) pathway. One of the major genes that influence both pathways as well as the regulation of the cell cycle (progression of cell division) is the tumour suppressor gene p53 [13,14]. Cancerous cells often suppress the p53 protein, upregulating anti-apoptotic BCL 2 family proteins. Suppression of p53 also results in inhibition of caspase enzymes such as caspase 3 and 7 that are effector genes responsible for executing apoptosis in cells [15].

Although, studies have demonstrated anti-proliferative and pro-apoptotic effects of different cereals, the mechanisms by which this activity occurs remain unclear [5,6,16,17]. This study aims to investigate the pro-apoptotic activity of whole grain cereal (rice, barley, oats and sorghum) phenolic extracts and the possible potential pathway to induce apoptosis in colorectal cancer cells. The results of this investigation contribute to the progressing notion of cereals as potential functional food that can aid in the reduction of cancer risk.

## 2. Results

### 2.1. Resazurin Assay

**Figure 1:**
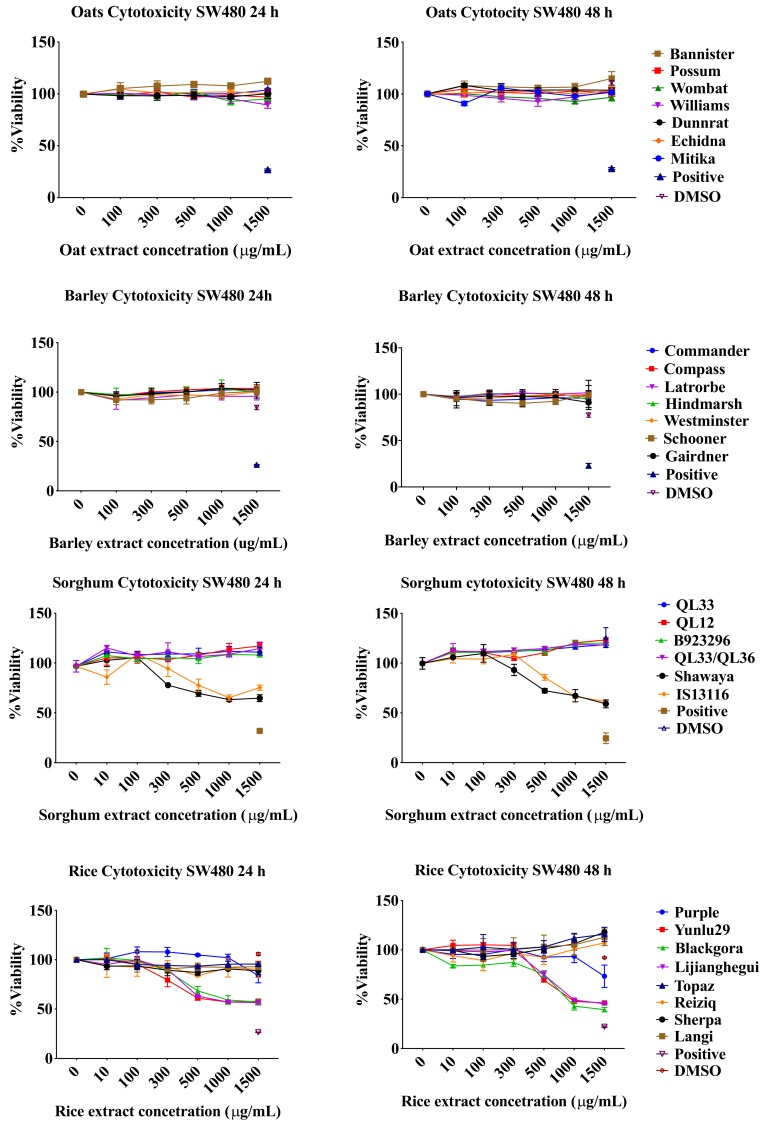
Cytotoxic effects of cereal phenolic extracts on colorectal cancer cell line SW480 at 24 h and 48 h. Results represent mean ± standard deviation (n = 3).

To test whether the various cereal extracts have an effect on the SW480 cells, a time dosage response cytotoxicity testing was conducted using resazurin dye. Colorectal cancer cells SW480 were treated with different varieties of rice, barley, oats and sorghum phenolic extracts at concentrations of 10, 100, 300, 500, 1000, 1500 µg/mL. Figure 1 exhibits the significant reduction in cancer cell viability in rice and sorghum extracts at 24 h and 48 h at dosages of 500 µg/mL and higher (*p* < 0.05). Extracts from the non-pigmented rice varieties did not affect the viability of cancer cells. The black pericarp sorghum variety Shawaya short black 1 and the brown pericarp sorghum variety IS13116 demonstrated inhibition of cell proliferation at a concentration of 500 µg/mL (*p* < 0.05). Red and white pericarp sorghum varieties did not affect cancer cell viability. Barley and oat phenolic extracts did not inhibit cell viability after 24 h or 48 h of treatment. Cereal extracts did not exhibit any significant cytotoxic effect at 24 h and 48 h on normal Fetal human colon (FHC) cell line at concentration of 500 µg/mL and lower. In some varieties of rice, barley and sorghum extracts minimal reduction in viability was exhibited at extremely high concentrations of 1000 µg/mL and/or 1500 µg/mL which is not attainable at physiological levels (Figure S1). In addition, this reduction could possibly be due to FHC cells sensitivity to changes in media constitution as DMSO of 3.74% (level present in the highest extract concentration) affected viability to a small degree.

### 2.2. Apoptosis Detection and Morphology

**Figure 2:**
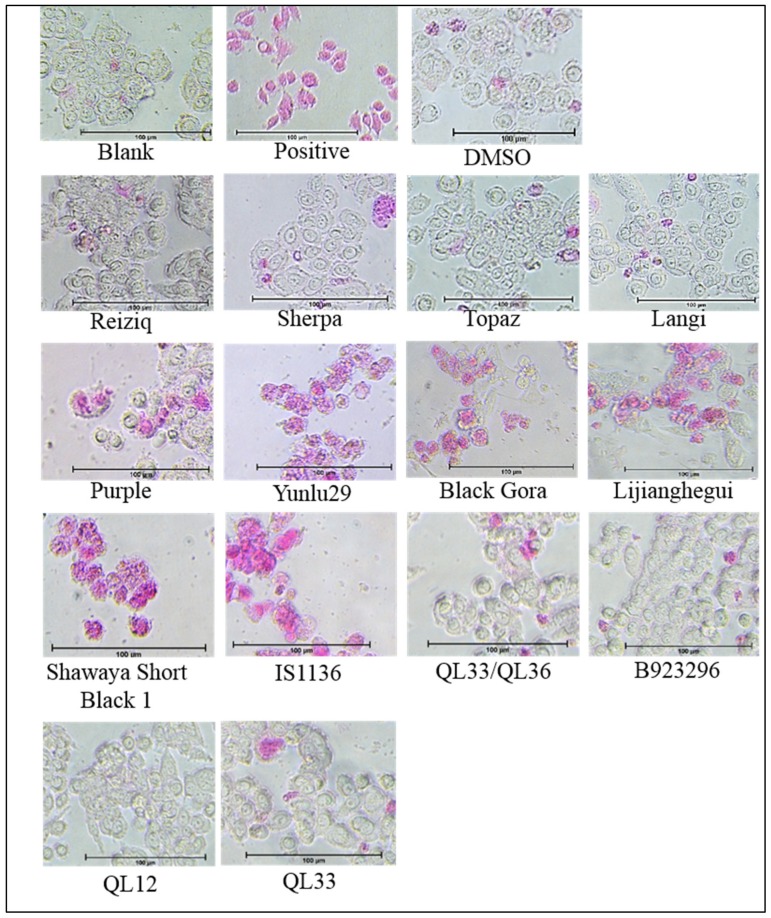
Apoptotic cells have compromised cell membrane which allows the APOPercentage dye (pink) to enter the cell. Retention of the dye after washing indicates apoptotic cell death in SW480 cells treated with rice and sorghum extracts at 24 h.

A morphological screening was preformed using the APOPercentage dye to identify if the cytotoxicity exhibited by selected cereal extracts was due to apoptosis. Sorghum varieties Shawaya short black 1 and IS11316, as well as the purple and red rice varieties, displayed pro-apoptotic effects with considerably high levels of dye retention, disruption of cell membrane integrity (Figure 2). Among the rice extracts Yunulu29 exhibited the highest level of pro-apoptotic activity, followed by Lijiangheui, Black Gora and Purple. Sorghum extracts, Shawaya short black and IS1136 were the most effective in inducing apoptosis. Similar to the cytotoxicity assay, no significant dye retention was observed in cells treated with extracts from non-pigmented rice, red and white sorghum varieties, barley or oat.

### 2.3. Annexin Analysis

**Figure 3:**
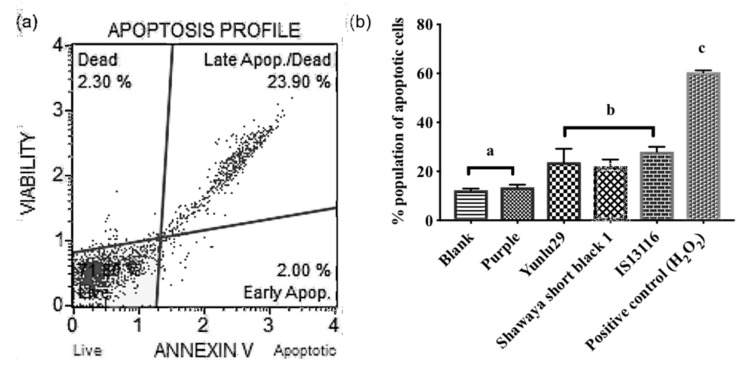
(**a**) Yunlu29 Muse plot of apoptosis induction (**b**) Proportion of pro-apoptotic activity using annexin assay in SW480 cells treated with rice and sorghum extracts. Bars with different letters represent significant difference in activity, results in (**b**) presented as mean ± standard deviation (n = 3).

An Annexin IV analysis was performed to confirm and quantify the pro-apoptotic activity exhibited in Section 2.2. Pro-apoptotic activity was quantified for the purple rice (Purple), red rice (Yunlu29), black sorghum (Shawaya short black 1) and brown sorghum (IS11316). Yunlu29, Shawaya short black 1 and IS11316 exhibited significant pro-apoptotic activity in comparison to the control (*p* < 0.05) (Figure 3).

### 2.4. Caspase and p53 Detection Assays

**Figure 4:**
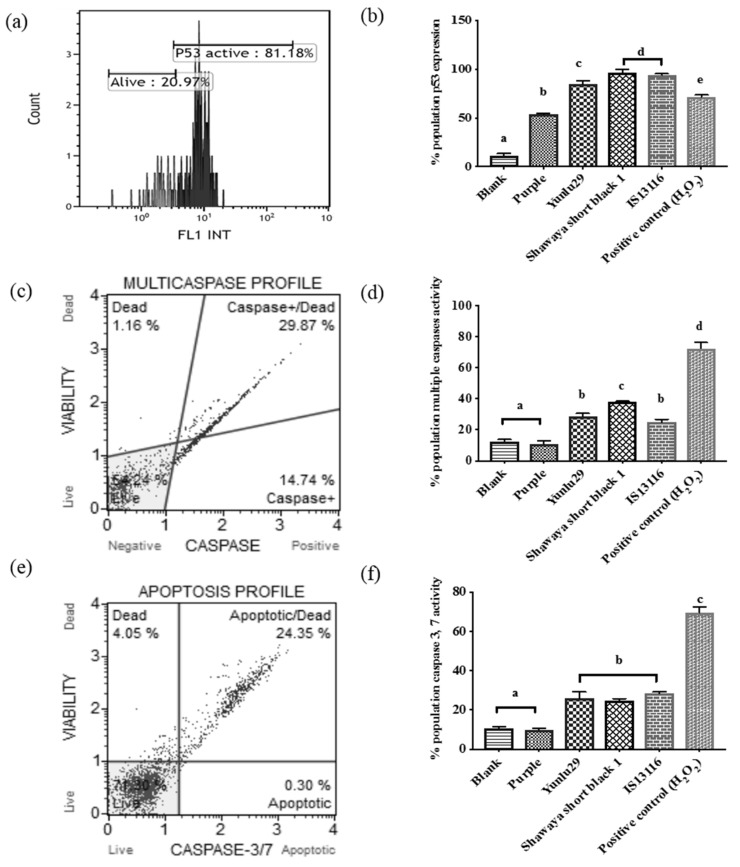
(**a**) Yunlu29 Flow cytometric histogram of p53 expression, (**b**) proportion of p53 expression, (**c**) Muse plot parameters for detection of multiple caspase activity (**d**) percentage of multiple caspase activity (**e**) Muse plot parameters for detection of caspases 3 and 7 (**f**) percentage of caspases 3 and 7 activity, in SW480 cells treated with rice and sorghum extracts. Bars with different letters represent significant difference in activity, results presented as mean ± standard deviation (n = 3).

To test the possible mechanism involved in the pro-apoptotic activity exhibited in SW480 cells when exposed to selected cereal extracts various assays were conducted. These assays included antibody labeled detection for expression of p53, multiple caspase and specifically caspase 3 and 7. Flow cytometric analysis with the Alexa Fluor 488 antibody showed increased expression of p53 in cells treated with phenolic compound extracts of Yunlu29, Purple, Shawaya short black 1 and IS11316 (*p* < 0.05) (Figure 4a,b). Further analysis using a Muse^®^ Cell Analyser demonstrated Yunlu29, Shawaya short black 1 and IS11316 to have considerable caspase activity involving one or more of 1, 3, 4, 5, 6, 7, 8 and 9 caspases as determined by the multiple caspase activity kit (*p* < 0.05) (Figure 4c,d). Shawaya short black was observed to have significantly higher caspase activation compared to Yunlu29 and IS11316. Yunlu29, Shawaya short black 1 and IS11316 also exhibited specific activation of caspase 3 and 7. However, cells treated with Purple rice extracts showed no significant caspase activity in either of the caspase assays.

## 3. Discussion

### 3.1. Anti-Proliferative Effect of Cereal Phenolic Extracts

In the current study barley and oat phenolic extracts did not exhibit significant anti-proliferative effect, while in rice and sorghum the pigmented varieties exhibited anti-proliferative activity starting at 500 µg/mL. Barley and oats when compared to rice and sorghum have considerably low phenolic contents and antioxidant activities [18,19]. However, studies have shown anti-proliferative effects of barley and oat phenolic extracts by treating cells with high concentrations of extract or prolong exposure for more than 48 h. Madhujith and Shahidi [20] demonstrated Canadian barley extracts to have anti-proliferative effects by treating human colorectal cancer cells (Caco-2 and HT-29) at 500 µg/mL for four days. Zhu, et al. [21] examined the anti-proliferative effects of free forms and bound forms of barley phenolic compounds. The study treated human liver cells (HepG2) with concentrations ranging from 0 to 200 mg/mL and found the lowest cytotoxic median effective dosage (EC_50_) to be at 66.4 mg/mL. A similar study with oats found anti-proliferative effects at an EC_50_ of 167.31 mg/mL in HepG2 cells [10]. Studies have also isolated specific bioactive compounds and examined their anti-proliferative effects. For example, protocatechualdehyde [22,23] in barley and avenanthramide (AVN) in oats [12,24,25] have been demonstrated to have anti-proliferative effects. Guo, et al. [12], determined that AVN C was toxic to colorectal cancer cell lines (HT-29, HCT 116, LS174TT and Caco-2) at 80–160 µM. While these treatments have achieved anti-proliferative effects on cancer cells, the concentrations applied may not be physiologically attainable post consumption of barley and oats.

Investigations of the anti-proliferative effects of rice have primarily used bran extracts and have reported a wide range of IC_50_ values. Banjerdpongchai, et al. [26] achieved anti-proliferative effects at 200 µg/mL with purple rice bran and Chatthongpisut, et al. [27] reported an IC_50_ of 12.63 with whole grain purple rice. On the other hand, the anti-proliferative effect of purple rice bran extract on leukaemia cells (APL) has been reported at 3.8 mg/mL at 24 h and 3.4 mg/mL at 72 h [28]. Forster, et al. [29] has demonstrated inhibition of proliferation at concentrations of 1, 3, 5 mg/mL using brown, red and purple rice varieties. In sorghum, similar anti-proliferative concentrations were observed in colon [30] and breast cancer cells [31]. In addition, these cytotoxic levels have been found to differ across cell lines, indicating a variation in the susceptibility of cancer cells [32]. Therefore, the anti-proliferative results observed in the current study are within the range of values reported in past literature.

Chemical profiling of the rice extracts in previous studies has demonstrated cyanidin-3-glucoside and peonidin-3-glucose to contain the highest free radical scavenging activity in purple rice [19]. It has been suggested that the cytotoxic effects exhibited by purple rice could be attributed to these anthocyanin polyphenols [3]. In red rice, the compounds likely to be responsible for the cytotoxic effect are a range of proanthocyanidins along with catechin derivatives. These bioactive compounds were observed to be present in significant amounts in the red rice varieties Yunulu29, Black gora and Lijianghegui [19]. The cytotoxic effects exerted by the extracts correlate with their overall total phenolic content and antioxidant activity. Similarly, sorghum varieties with higher total phenolic content and antioxidant activity generally exhibited greater levels of cancer cell cytotoxicity. In addition to proanthocyanidins, sorghum extracts have been identified to contain 3-deoxyanthocyanin (3DXA) a derivative form of anthocyanin and have been linked to sorghum anti-cancer potential [9,17,33,34,35]. However, unlike rice, sorghum phenolic antioxidant activity is not dominated by a single compound and high antioxidant activity is a cumulative effect [36].

### 3.2. Mechanism of Apoptotic Effect

Apoptotic effect was identified using the APOPercentage dye and morphological observation. Both rice and sorghum extracts induced apoptosis in the colon cancer cells. This was further confirmed via Annexin facilitated quantification of apoptotic activity. Shawaya short black, IS11316 and Yunlu29 exhibited higher apoptotic activity compared to Purple rice. Further analysis showed that Yunlu29, Shawaya short black and IS11316, increased the expression of the tumour regulator protein p53, multiple caspases and the effector caspase 3 and 7 when compared to the control. Although cancerous cells such as SW480 have an underlying impaired p53 function, which allows for uncontrolled proliferation and tumour progression, the cereals extracts demonstrated the potential to increase its expression. In spite of the potential of the cereal extracts contributing to the increased expression, further mechanistic studies are warranted to confirm restoration of the tumour suppressor functionality. Purple rice also expressed p53 levels higher than the control, however, caspase activation was not detected, and neither was significant level of apoptotic activity detected. The decreased cell viability observed in the cytotoxicity assay could be due to cell cycle arrest which would halt cellular proliferation consequently indicating low cell viability in the cytotoxicity assay. Alternatively, employing a caspase-independent pathway such as mitochondrial membrane potential loss [37] may have been responsible for cell death. Apoptotic induction via p53 expression or activation of caspase activity is one possible pathway employed by cereal extracts on SW480 cells, which may vary depending on cell types and tumour mutations. Further research is warranted to gain a comprehensive understanding of all the potential pathways of apoptotic induction by cereal extracts.

**Figure 5:**
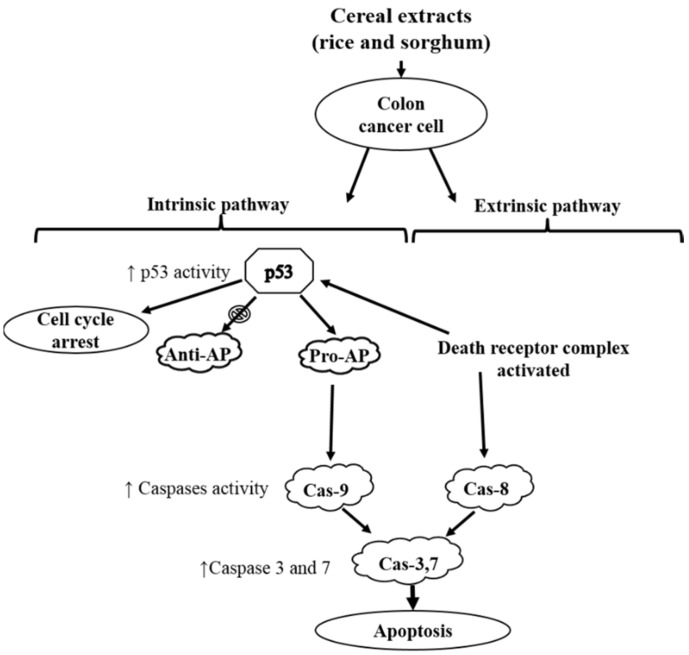
Potential activity pathways for apoptosis induction resulting from cereal phenolic compound exposure (↓ downregulation, ↑ upregulation, Caspase (Cas), pro-apoptotic protein (Pro-AP), anti-apoptotic protein (anti-AP)). Modified from Rao, et al. [32].

A number of apoptotic induction-related mechanisms have been proposed in the literature, which describes results that are similar to those found in the current study [4,26]. Kong, et al. [38] investigated the apoptotic potential of rice phenolic compounds and found that the mechanism of action involved downregulation of the BCL-2 protein, an anti-apoptotic protein which is regulated by p53. Another proposed mechanism of action was activation of caspase 9 and 3 [26]. Investigations on sorghum phenolic extracts, specifically 3DXA, have been associated with the activation of p53 as well as the subsequent upregulation of pro-apoptotic proteins and downregulation of anti-apoptotic proteins [31,32]. The current study concurs the mechanism of action in inducing apoptosis in colorectal cancer cells by rice (red varieties) and sorghum (black and brown varieties) is likely to be by upregulation of the p53 protein activating its functionality. Consequently, a cascading effect resulting in activated caspase 3 and 7 is initiated, ultimately leading to cell death by apoptosis. Another possible anti-proliferative mechanism is cell cycle arrest as demonstrated with the Purple rice activity, this can be tested with further analysis on cell cycle stages. Therefore, cereals such as pigmented rice, black and brown sorghum have substantial impacts on cancer cell viability, reducing its proliferation via induction of the apoptotic pathway. These potential mechanisms are illustrated in Figure 5.

## 4. Materials and Methods

### 4.1. Materials

Apo transferrin, Dimethyl sulphoxide (DMSO), Dulbecco’s modified eagle’s medium (DMEM), foetal bovine serum (FBS), penicillin-streptomycin, phosphate buffer saline (PBS), trypsin and hydrogen peroxide (H_2_O_2_), resazurin, fixation buffer and stain buffer were obtained from Sigma-Aldrich (St. Louis, MI, USA). Normal FHC cells, DMEM: F12 Medium (ATCC 30-2006), 4-(2-hydroxyethyl)-1-piperazineethanesulfonic acid (HEPES), hydrocortisone, cholera toxin, insulin, human recombinant epidermal growth factor (EGF) were sourced from ATCC distributor In Vitro Technologies Pty Ltd. (Victoria, Australia). ApoPercentage™ was supplied by Biocolor (Belfast, UK). Extraction solvents including acetone and acetic acid were sourced from Chem Supply Pty Ltd. (South Australia, Australia). Annexin V assay, multiple caspase assay and caspase 3/7 assay were sourced from Merck (Victoria, Australia). SW480 cells were provided by Dr Vinod Gopalan from Griffith University, Australia [39], which were purchased from ATCC. Rice grains were sourced from field trials conducted by the New south Wales Department of Primary Industries, Yanco, Australia. Rice samples included Purple (Purple), red (Yunlu29, Lijianghegui, Black gora) and non-pigment varieties (Reiziq, Sherpa, Langi and Topaz). Barley and oat samples were sourced from the National Variety Trials commissioned by the Grains Research and Development Corporation, Australia. Barley varieties included Commander, Compass, Hindmarsh, Latrobe, Westminster, Gairdner and Schooner. Oats varieties included Bannister, Dunnrat, Echidna, Possum, Mitika, Williams, Wombat and Yallara. Sorghum samples were obtained from glasshouse trails conducted at Curtin University, Western Australia, Australia. Pigmented varieties of sorghum were black (Shawaya short black 1), brown (IS11316), red (QL33, QL33/QL36 and B923296) and the non-pigmented (white) variety was QT12.

### 4.2. Preparation of Extracts

Whole grain samples of oats, rice and sorghum were used for phenol compound extraction. Barley samples also included the husk as it is tightly attached to the endosperm. Samples were ground using a Perten Laboratory Mill 3000 (Hägersten, Sweden) and sieved 100% through a 0.5 mM sieve plate. Extraction procedure followed methodology reported in previous publications [18,19]. Details of phenolic compound composition and antioxidant activity have been reported in References [18,19,40], for rice, oats and barley respectively. Sorghum chemical profile has been reported by Rao, et al. [36] and variation with methanol extraction has been reported by Wu, et al. [41]. The final extracts were resuspended in diluted DMSO. Freeze-dried extracts were reconstituted to stocks of 20 mg/mL in 50% DMSO and stored at −20 °C.

### 4.3. Cell Culture

SW480 colorectal cancer cell lines were grown in DMEM and maintained as a monolayer. DMEM media was supplemented with 10% (*v*/*v*) FBS and 1% (*v*/*v*) penicillin-streptomycin. The normal colon cell line FHC was cultured in DMEM: F12 Medium (ATCC 30-2006) supplemented with HEPES, hydrocortisone, cholera toxin, insulin, transferrin, human recombinant EFG and 10% FBS. The cultures were grown in an incubator at 37 °C in a humidified atmosphere of a 5% (*v*/*v*) CO_2_ in the air. Incubation of cells during assays was conducted in the aforementioned conditions unless stated otherwise.

### 4.4. Resazurin Cytotoxicity Assay

A time/dosage response assay was conducted to test cytotoxicity. Extracts were diluted to concentrations of 10, 100, 300, 500, 1000, 1500 µg/mL prior to testing. SW480 cells were cultured in 96 well plates at a cell density of 5 × 10^4^ per well. After 24 h of incubation, cells were washed with PBS and incubated with the various extract preparations. An additional plate was also prepared as a blank (extracts with no cells) to compensate for extract colour. A negative control of plain media, positive control of 10 mM hydrogen peroxide and DMSO control of 3.7% (the maximum level present in reconstituted extracts) were also included. The experiment was replicated on FHC cells to test for cytotoxic effect of cereal extracts on normal cells. After 24 and 48 h treatments, 100 µL of resazurin dye was added. The dye was prepared according to Ataollahi, et al. [42] and Callcott, et al. [43]. After the reaction incubation period, absorbance was measured at 570 and 600 nm and calculated as described by Ataollahi, et al. as viability percentage [42]. All treatments were performed and measured three times (n = 3).

### 4.5. APOPercentage™ Assays

To identify the cause of SW480 cell death, apoptosis induction and the permeability of cell membranes was assessed using the APOPercentage kit and protocol. Briefly, SW480 cells were seeded as reported in Section 4.3, washed and treated with the cereal phenolic extracts. Barley and oat phenolic extracts at a concentration of 1500 µg/mL were used, while rice and sorghum extracts of 500 µg/mL were used due to their higher cytotoxicity as determined by the cytotoxicity assay. Cells were incubated for 24 h and 30 min prior to completion, cells were treated with fresh media containing cereal extracts and 5% *v*/*v* APOPercentage dye. Normal media was employed as a negative control and 10 mM hydrogen peroxide was employed as a positive control, impact of DMSO used in suspension of extracts was also tested at the highest concertation of 3.7%. After 30 min of incubation, reagent B was removed, and cells were washed twice with PBS. Morphological observation dye retention and visual assessment were recorded using a Nikon Eclipse Ti compound microscope (Tokyo, Japan) and imaging was with an assembly consisting of a Nikon intensilight C-HGFI illuminator, a Digital Sight DS-U3 camera controller, a DS-Fi2 camera and the NIS-Elements imaging software (Melville, New York, NY, USA).

### 4.6. Muse Annexin Assay

The Muse^TM^ Annexin V and death cell assay antibody binds to externalized PS indicating, early apoptotic, late apoptotic and dead cells. Muse^TM^ Annexin assay was conducted using the user guide on the Muse^®^ Cell Analyser (Merck, Victoria, Australia). SW480 cells were cultured in 24 well plates at a concentration of 1 × 10^5^ cell/mL and treated with the lowest effective cytotoxic concentration (500 µg/mL) of phenolic compound extracts from Purple, Yunlu29, Shawaya short black 1 and IS11316. The assay involved detaching treated adherent cells with trypsin. Cells were resuspended in media containing 1% FBS at 1 × 10^5^ cell/mL concentration. Then 100 µL of cell suspension was mixed with 100 µL of warmed Muse™ Annexin V antibody. The sample was incubated for 20 min in the dark at room temperature and then measured using Muse cell analyser as population percentage of live, early apoptotic, late apoptotic and dead cells. All treatments were performed in triplicates and results were acquired by Muse^®^ Cell Analyser.

### 4.7. Muse Multiple Caspase Assay

The Muse^TM^ Multi caspase kit was used to measure the activity of caspases 1, 3, 4, 5, 6, 7, 8 and 9 by employing a VAD-peptide in combination with a cell viability dye (7-AAD). The membrane VAD-peptide is composed with a fluorescent group and an FMK moiety, FLICA (Fluorescent-labeled inhibitor of Caspase). This conformation allows the VAD-peptide to permeate cells and bind to active caspases, the resultant fluorescence is directly proportional to caspase activity. SW480 ells were cultured in 24 well plates at a concentration of 1 × 10^5^ cell/mL in cell culture media as stated above. After 24 h of incubation, cells were washed with PBS and treated with the lowest cytotoxic concentration of 500 µg/mL phenolic extracts of Purple, Yunlu29, Shawaya short black 1 and IS11316. Afterwards, cells were washed, dislocated and resuspended in media containing 1% FBS at a concentration of 1 × 10^5^ cell/mL. A 50 µL cell suspension was mixed with 5 µL of Muse^TM^ Multi caspase working solution reagent, lightly vortexed and incubated for 30 min at 37 °C in a humidified atmosphere containing 5% (*v*/*v*) CO_2_. After incubation, 150 µL of 7-AAD dye working solution was added to the mixture and the mixture was incubated in the dark for 5 min. Following incubation, measurements were obtained using the Muse cell analyser and compared to a blank and a 10 mM H_2_O_2_ positive control. The measurement was recorded as population percentage of no caspase activity and live cells (live), caspase activity in live cells (caspase +), caspase activity in dead cells (caspase +/ dead), and death/ necrotic cells (dead). All treatments were performed three times.

### 4.8. Muse Caspases 3/7 Assay

Caspases 3 and 7 play an important role in the cascading signal of cellular apoptosis, specifically, cleaving structural proteins. Caspase 3/7 activity was measured using the Muse Caspase 3/7 kit as outlined in the kit protocol. The Muse^TM^ Caspase-3/7 kit contains a DNA binding dye that is attached to DEVD peptide, this form inhibits the dye from binding to DNA. In the presence of active caspase 3/7, the dye is cleaved from the DEVD peptide and is able to translocate to the nucleus, where binding with DNA results in high fluorescence which is measured with the Muse^TM^ Cell analyser. For the assay, SW480 cells were cultured as stated in Section 4.6. A 50 µL cell suspension with cell concentration of 1 × 10^5^ cell/mL was mixed with 5 µL of Muse^TM^ Multi caspase working solution reagent working solution reagent, lightly vortexed and incubated for 30 min at 37 °C in a humidified atmosphere of a 5% (*v*/*v*) CO_2_ in the air. After incubation 150 µL 7-AAD dye working solution was added to the mixture and incubated in the dark for 5 min. Following incubation, measurements were obtained using Muse cell analyser, the set up included a blank and a 10 mM H_2_O_2_ positive control. The measurement was recorded as population percentage of cells undergoing apoptosis and exhibiting caspase 3 and 7 activity. The Muse plot included quadrants of live cells with no caspase 3 and 7 activity (live), caspase 3 and 7 activity in live cells (apoptotic), caspase 3 and 7 activity in dead cells (apoptotic/ dead), and death/ necrotic cells (dead). All treatments were performed three times.

### 4.9. p53 Expression in SW480 Cells

Cells were cultured as stated in Section 4.6 at 1 × 10^5^ cell/mL and assayed using Alexa Fluor 488 antibody from BD Life Sciences (Ryde, Australia). Detached cells were suspended in 500 µL of room temperature fixation buffer (formaldehyde) and incubated in the dark at room temperature for 10 min. After incubation, cells were centrifuged and washed with 500 µL stain buffer and centrifuged. After centrifugation cells were lysed with 500 µL of ice-cold permeation buffer and incubated for 30 min on ice. Cells were then centrifuged and washed with 500 µL of stain buffer and centrifuged. Washed cells were suspended in 100 µL of stain buffer, labeled with 10 µL of Alexa Fluor 488 and incubated for 30 min at room temperature in the dark. After incubation, excess dye was washed off cells with 500 µL of stain buffer. Pelleted cells were suspended in 500 µL of stain buffer and analysed on a Beckmann Coulter flow cytometer (Lane Cove, Australia). Florescence emission of labeled p53 was recorded as population percentage of p53 expression. All treatments were performed three times.

### 4.10. Statistical Analysis

Statistical analysis was conducted by means of a one-way and two-way analysis of variance (ANOVA), followed by posthoc Tukey’s multiple comparisons test using GraphPad Prism 7 software (GraphPad Software Inc, California, CA, USA). The results are reported as mean ± standard deviation. Statistical significance was determined at a level of *p* < 0.05.

## 5. Conclusions

Phenolic extracts derived from cereals were observed to exhibit an apoptotic effect on colorectal cancer cells with significant varietal variation. A parallel between high antioxidant activity and phenolic content was associated with substantial anti-cancer activity in pigmented varieties of rice and sorghum. The proposed mechanism of anti-cancer activity exhibited by the phenolic extracts in the current study is believed to be via p53 activity downstream and/or a direct effect on executioner caspases 3 and 7, inducing cell death in colorectal cancer cell lines by apoptosis. However, this is only one possible mechanism, cereal extracts may have more than one pathway of inducing apoptosis. Furthermore, replicating this investigation on other forms of cancer will also provide a better understanding of the different possible apoptotic induction pathways, as the current assays only investigated one form of cancer. Further investigations such as studying the effect on specific pro and anti-apoptotic genes, mitochondrial potential, cytochrome c expression and cell cycle analysis can also help determine if the apoptotic pathway is intrinsic or extrinsic. These investigations can also confirm if the expression of the p53 protein by cereal extracts translates to activation of its functionality. In addition, in vivo studies are required to better understand the effect of phenolic compounds and its interaction with other metabolites have on cancerous cells and to what extent these beneficial effects are manifested in the human body when consumed.

## Supplementary Materials

Figure S1. Cytotoxic effects of phenolic extracts on normal colorectal cells FHC at 24 h and 48 h, results represent mean ± standard deviation (n = 3).

## Abbreviations

Dimethyl sulphur dioxide (DMSO), Dulbecco’s modified eagle’s medium (DMEM), foetal bovine serum (FCS), phosphate buffer saline (PBS), hydrogen peroxide (H_2_O_2_) and half maximal inhibitory concentration (IC_50_).
