# Supplementary material for: Apoptosis Induction Pathway in Human Colorectal Cancer Cell Line SW480 Exposed to Cereal Phenolic Extracts
Source: Molecules. 2019 Jul 4;24(13):2465. doi: 10.3390/molecules24132465 (PMC6651285; doi:10.3390/molecules24132465)
Supplement: Supplementary file 1 [file molecules-24-02465-s001.pdf]

molecules

MDPI

Article

Apoptosis Induction Pathway in Human Colorectal Cancer Cell Line SW480 Exposed to Cereal Phenolic Extracts

Shiwangni Rao [1], Kenneth Chinkwo [1,*], Abishek Santhakumar [1], Stuart Johnson [2] and Christopher Blanchard [1]

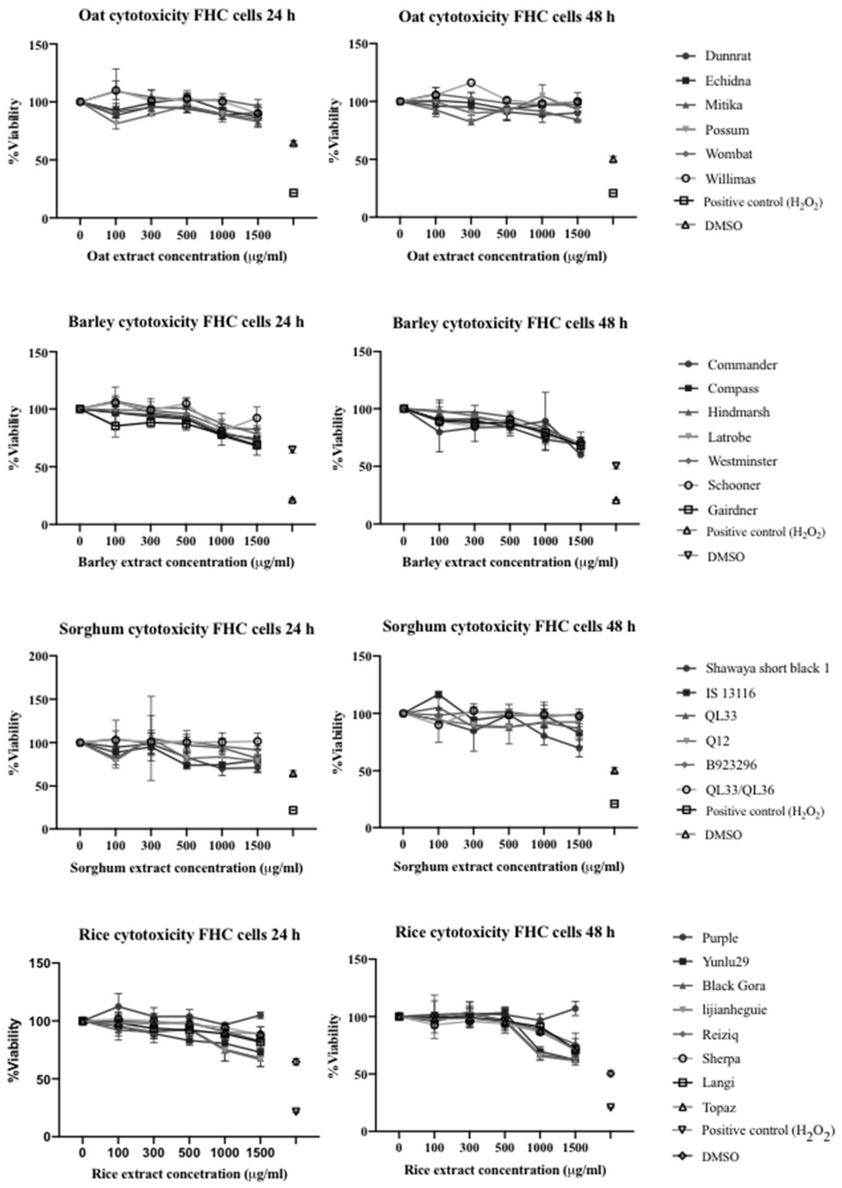

Figure 1. Cytotoxic effects of phenolic extracts on normal colorectal cells FHC at 24 h and 48 h, results represent mean ± standard deviation (n = 3).
